# Utilizing visual symptoms to distinguish dry eye from glaucoma, cataract, and suspect glaucoma patients: a cross-sectional study

**DOI:** 10.1186/s12886-023-03219-2

**Published:** 2024-01-09

**Authors:** David X. Zhao, Michael Quintero, Aleksandra Mihailovic, Esen Akpek, Sezen Karakus, Lee Guo, Pradeep Y. Ramulu

**Affiliations:** 1 Johns Hopkins Wilmer Eye Institute, Baltimore, MD USA; 2 Wilmer Eye Institute, Johns Hopkins Medical Institutions, 600 N. Wolfe St., Wilmer 129, Baltimore, MD 21287 USA

**Keywords:** Dry eye, Glaucoma, Cataract, Visual symptoms

## Abstract

**Background:**

The diagnosis of dry eye and other common ophthalmological conditions can be supported using patient reported symptoms, which is increasingly useful in contexts such as telemedicine. We aim to ascertain visual symptoms that differentiate dry eye from cataract, glaucoma, or glaucoma suspects.

**Methods:**

Adults with dry eye, glaucoma, cataract, and suspected glaucoma (controls) completed a questionnaire to rate the frequency and severity of 28 visual symptoms. Univariate, followed by multivariable logistic regression with backward stepwise selection (*p* < 0.05), determined the individual symptoms and set of symptoms best distinguishing dry eye from each of the other conditions.

**Results:**

Mean age of 353 patients (94 glaucoma suspect controls, 79 glaucoma, 84 cataract, and 96 dry eye) was 64.1 years (SD = 14.1); 67% were female and 68% White. Dry eye patients reported more frequent light sensitivity (OR = 15.0, 95% CI = 6.3–35.7) and spots in vision (OR = 2.8, 95% CI = 1.2–6.3) compared to glaucoma suspect controls. Compared to glaucoma patients, dry eye patients experienced more frequent light sensitivity (OR = 9.2, 95% CI = 2.0–41.7), but less frequent poor peripheral vision (OR = 0.2, 95% CI = 0.06–0.7), difference in vision between eyes (OR = 0.09, 95% CI = 0.01–0.7), and missing patches of vision (OR = 0.06, 95% CI = 0.009–0.3). Compared to cataract patients, dry eye patients reported more frequent spots in vision (OR = 4.5, 95% CI = 1.5–13.4) and vision variability across the week (OR = 4.7, 95% CI = 1.2–17.7) and were less likely to report worsening vision (OR = 0.1, 95% CI = 0.03–0.4) and blindness (OR = 0.1, 95% CI = 0.02–0.8).

**Conclusion:**

Visual symptoms may serve as a complementary tool to distinguish dry eye from various ocular conditions, though the symptoms that best distinguish dry eye differ across comparisons. Differentiating how patients visually perceive common eye diseases may be used in a variety of clinical settings to rule out specific conditions.

**Supplementary Information:**

The online version contains supplementary material available at 10.1186/s12886-023-03219-2.

## Introduction

There has been a growing emphasis on incorporating patients' input in the management of eye disease. One advantage of acquiring patient input is in disease diagnosis, by enabling patients to express their symptoms in the context of clinical information used by physicians to help identify the condition accounting for those symptoms [1, 2]. Patient-reported visual symptoms can be especially helpful during phone calls, or when identifying distinct eye conditions during telemedicine visits, which have significantly increased since the onset of the COVID-19 pandemic [3]. Symptoms can also be a helpful adjunct in clinical visits when ocular examination is available. This is especially important for conditions such as dry eye, where the symptoms that characterize dry eye and objective measurements through direct examination (e.g. ocular surface staining [OSS], tear break-up time [TBUT], tear osmolarity, Schirmer’s test) are poorly correlated [4, 5]. Variation in corneal sensitivity can also lead to discrepancy between the signs and symptoms of dry eye [6]. Additionally, the objective findings of dry eye are often variable over time, non-specific and can be found in normal eyes as well. Therefore, the diagnosis of dry eye has largely relied on patient-reported symptoms.

Dry eye patients commonly complain of tactile symptoms pertaining to pain and discomfort of the eyes; visual symptoms like light sensitivity, glare, and blurry vision are also prominent in dry eye condition [7–13]. However, it is unclear to what extent various visual symptoms differentiate dry eye from other eye conditions. Previous studies have shown that dry eye commonly co-exists with cataracts (40–80% of patients scheduled to have cataract surgery) [14, 15] and glaucoma (20–59% of glaucoma patients) [16, 17]. Past work in our group also suggests that patient-reported symptoms can aid in distinguishing conditions such as glaucoma and cataracts [18]. The ability to distinguish dry eye from conditions like glaucoma and cataract based on the incorporation of visual symptom assessment could potentially improve patient communication and appropriately direct treatment toward the proper disease.

To our knowledge, the visual symptoms that differentiate dry eye patients from cataract or glaucoma patients have not been ascertained. The aims of this study are to determine: [1] the most common visual symptoms reported by dry eye, glaucoma, cataract, and suspect glaucoma (control) patients, (2) the individual visual symptoms which distinguish significant dry eye from glaucoma suspects, glaucoma, as well as from patients with visually significant cataract, (3) the set of symptoms which best predicts dry eye diagnosis versus one of the other study diagnoses, and (4) symptoms which are not specific, and generally unable to distinguish dry eye from other conditions. We hypothesize that there are individual symptoms commonly caused by dry eye, such as light sensitivity and glare, that can help differentiate dry eye from cataract, glaucoma, and persons without disease.

## Methods

### Study participants

This is a cross-sectional study of patients with clinically-significant dry eye, suspected glaucoma (controls), glaucoma, or cataracts. Study participants older than the age of 18 were recruited from the Wilmer Eye Institute at Johns Hopkins Hospital between June 2019-December 2022. The study procedures were explained to participants and informed consent was obtained. The study and its procedures were approved by the Johns Hopkins Institutional Review Board and performed in accordance with the Helsinki Declaration of 1975.

Clinically significant dry eye patients were included if they had: (1) total OSS score of 5 or more in both eyes, and (2) visual acuity (VA) of 20/30 or better in both eyes. Suspect glaucoma patients (controls) were included if they had (1) visual field (VF) mean deviation (MD) of -4 dB or better in both eyes, and (2) chart diagnosis of primary open-angle glaucoma suspect or primary angle-closure glaucoma suspect. Glaucoma patients were included if they had: (1) VF MD of -5 decibels (dB) or worse in both eyes, and (2) chart diagnosis of primary open-angle glaucoma (including pseudoexfoliation glaucoma) or primary angle-closure glaucoma. Cataract patients were included if they had: (1) VA of 20/30 or worse in both eyes, and (2) chart diagnosis of bilateral cataracts with severity requiring surgical intervention.

All participants underwent slit-lamp examination and fundoscopy, and were excluded if any visually significant eye disease (except that which qualified them for their study group) was diagnosed, including pathologies of the cornea, retina, lens, and ocular nerves. Dry eye patients were excluded if they had any previous eye surgery, with the exception of uncomplicated cataract surgery. Glaucoma suspect control patients were excluded if they had (1) VA of 20/40 or worse in either eye, or (2) cataracts of suspected clinical significance. Clinically significant cataract was ruled out in the control, glaucoma, and dry eye groups by the absence of a cataract treatment plan (e.g. surgery) in the patient chart, or mention of visual significance of cataract (even if the patient declined surgery). Cataract patients were excluded if they had prior cataract surgery in either eye. As the three non-dry eye groups were not formally evaluated with regards to their ocular surface features, no individuals were excluded based on the possibility of co-existing dry eye. Patients were also excluded if they were non-English speakers or had mental or general health conditions that prevented them from answering questions.

Chart diagnoses, Sjögren’s International Collaborative Clinical Alliance (SICCA) OSS scores (for dry eye patients), [19] VF MD (for glaucoma patients and glaucoma suspect controls), and VA measurements were obtained from the most recent clinic visit. Monocular VF testing was performed using a Humphrey Visual Field Analyzer II or III (Carl Zeiss Meditec, Inc) using any version of the Swedish Interactive Testing Algorithm (SITA). Snellen best-corrected VA was converted to logarithm of the minimum angle of resolution (logMAR) for analysis. Patient demographic information, prescribed medications taking, and ocular history were collected from the electronic medical record chart notes.

### Questionnaire

Patients were consented and completed a questionnaire asking about various visual symptoms within one month of the office visit from which inclusion and exclusion criteria were determined. Participants were asked to describe their vision and to rate the frequency ([1] never, [2] rarely, [3] sometimes, or [4] very often) and severity ([1] not at all, [2] mild, [3] moderate, or [4] severe) of 28 previously-described visual symptoms derived from prior questionnaires or symptom banks [20–29]. The full questionnaire is available as supplementary information. Glaucoma suspect and glaucoma patients completed the questionnaire on their own unless the patient had difficulty reading the questionnaire. In that case, a research coordinator orally administered the questionnaire. All cataract and significant dry eye patients had the questionnaire administered to them orally over the telephone due to COVID-19 restrictions.

### Statistical analysis

Demographic data (sex, race, education level, employment, number and class of prescription eye drop medications taking) were compared across all disease groups using chi-squared or Fisher’s exact test. Age and VA (logMAR) was compared across all groups using the Kruskal–Wallis test. The distribution of symptom frequency rating (never, rarely, sometimes, very often) and severity rating (not at all, mild, moderate, severe) were compared between all disease groups using chi-squared testing. Frequency of symptoms and severity of symptoms were binarized between the absence of a symptom and a symptom reported at any frequency or severity. Univariate logistic regression was performed with the outcome being dry eye diagnosis versus one of the other disease states, and the independent variable being the binarized frequency or severity response for each of the 28 visual symptoms. Significant associations from univariate analysis were carried forward into multivariable analysis. Highly collinear variables were removed from the model until mean of model variance inflation factors was < 2.5 and all variables had a variance inflation factor of < 5. Backward stepwise selection (*p* < 0.05) was performed on the remaining variables along with the demographic variables to determine the set of visual symptoms that best distinguishes dry eye from each other state. These final models were compared to a model containing only demographic variables using a likelihood ratio test to determine if the visual symptom variables significantly contribute to differentiating between the diseases. Patients with missing symptom variable responses were excluded from logistic regressions. fivefold cross-validation was used to calculate the mean area under the curve (AUC) for the receiver operator curve (ROC) of the ability of the final model to predict a dry eye diagnosis from one of the other study conditions [30]. Sensitivity and specificity were denoted at a predicted probability level of 0.5 or greater. All statistical analysis were performed on Stata v17.0 (StataCorp, College Station, Texas) [31]. Statistical significance was defined as *p* < 0.05 for all analyses. The datasets generated during and/or analyzed during the current study are not publicly available, but are available from the corresponding author on reasonable request.

## Results

### Demographics

Three hundred fifty three patients were consented and completed the questionnaire, including 96 dry eye, 94 suspected glaucoma, 79 glaucoma, and 84 cataract patients. Age differed significantly across all groups (*p* = 0.001), with cataract patients being the oldest (mean age 73.2 years, standard deviation [SD] = 8.7) and dry eye patients being the youngest (mean age 55.2 years, SD = 14.4) (Table 1). The groups differed significantly in terms of sex (*p* < 0.001), with dry eye patients having the greatest proportion of females (91.7%), and glaucoma patients the least (49.4%). VA in the better eye and worse eye also differed significantly across disease groups (*p* < 0.001 for both), with dry eye patients having the best VA and cataract patients the worst. The median number of prescription eye drops differed significantly across all disease groups. Three glaucoma suspect, two glaucoma, and four cataract patients were on prescription dry eye topical treatments. One dry eye patient was taking a glaucoma prescription eye drop but did not have a diagnosis of glaucoma. Study groups also differed by employment status, race, and education (*p* < 0.01 for all).

**Table 1:** Population characteristics of study participants

Demographics	All Patients (*n* = 353)	Dry Eye (*n* = 96)	Glaucoma Suspects (*n* = 94)	Glaucoma (*n* = 79)	Cataract (*n* = 84)	*P* Value
Age (years), mean (SD)	64.1 (14.7)	55.2 (14.4)	61.0 (15.2)	69.0 (12.2)	73.2 (8.7)	0.001
Female, n (%)	235 (66.6)	88 (91.7)	61 (64.9)	39 (49.4)	47 (56.0)	< 0.001
Employed, n (%)^a^	152 (43.1)	52 (54.2)	49 (55.1)	24 (34.3)	27 (32.1)	0.001
Race, n (%)						< 0.001
White	241 (68.3)	72 (75.0)	52 (55.3)	42 (53.2)	75 (89.3)	
African American	68 (19.3)	13 (13.5)	25 (26.6)	27 (34.2)	3 (3.6)	
Asian or Pacific Islander	28 (7.9)	9 (9.4)	12 (12.8)	6 (7.6)	1 (1.2)	
Other	16 (4.5)	2 (2.1)	5 (5.3)	4 (5.1)	5 (6.0)	
Education, n (%)						0.008
Less than high school	10 (2.8)	0 (0)	2 (2.1)	6 (7.6)	2 (2.4)	
High school	37 (10.5)	7 (7.3)	8 (8.5)	12 (15.2)	10 (11.9)	
Some college	68 (19.3)	12 (12.5)	25 (26.6)	16 (20.3)	15 (17.9)	
Bachelor's degree	76 (21.5)	31 (32.3)	17 (18.1)	15 (19.0)	13 (15.5)	
More than Bachelor's	162 (45.9)	46 (47.9)	42 (44.7)	30 (38.0)	44 (52.4)	
VA (logMAR), median (IQR)						
Better eye	0 (0–0.18)	0 (0–0)	0 (0–0)	0.10 (0–0.30)	0.30 (0.18–0.40)	< 0.001
Worse eye	0.18 (0.10–0.40)	0 (0–0.097)	0.10 (0–0.18)	0.30 (0.18–0.60)	0.48 (0.35–0.75)	< 0.001
Prescription eye drops, median (IQR)	1 (0–2)	1 (1–2)	0 (0–1)	2 (1–3)	0 (0–0)	< 0.001
Taking prescription glaucoma eye drops, n (%)^b^	93 (26.3)	1 (1.0)	22 (23.4)	70 (88.6)	0 (0)	< 0.001
Taking prescription dry eye drops, n (%)^c^	94 (26.6)	85 (88.5)	3 (3.2)	2 (2.5)	4 (4.8)	< 0.001

*SD* standard deviation, *IQR* interquartile range, *logMAR* logarithm of the minimum angle of resolution

^a^14 patients were missing employment status, therefore proportions of employed patients for all patients, glaucoma, and glaucoma suspects were calculated out of 339, 70, and 89, respectively

^b^Glaucoma eye drop medications include: Latanoprost, Dorzolamide, Timolol, Brimonidine, Travoprost, Carteolol, Cosopt, Bimatoprost, Brinzolamide, Netasudil

^c^Dry eye topical treatments include: Cyclosporine (Restasis, Cequa), Xiidra, autologous serum eye drops (Vital Tears, NovaTears), Loteprednol (Lotemax)

### Most common visual symptoms among different disease groups

**Fig. 1:**
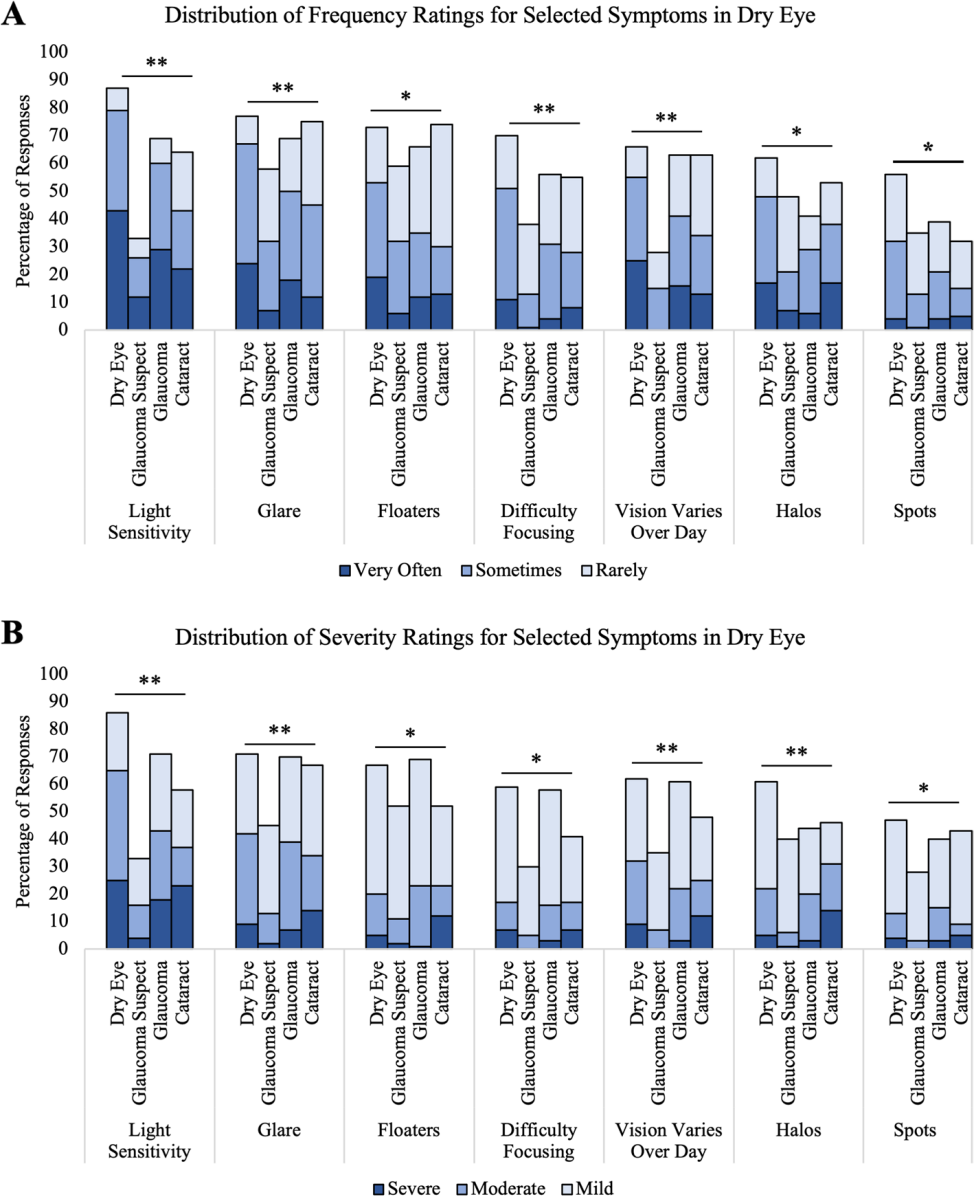
**A** Distribution of frequency responses for the seven symptoms most commonly experienced among at least 50% of dry eye patients. **B** Distribution of severity responses for the seven symptoms most commonly experienced among at least 50% of dry eye patients. Chi-squared test was used to compare the distribution of responses across all disease groups. *p < 0.05, **p < 0.001

Some symptoms were common amongst all groups (occurring in more than 50% of respondents in each group), including blurry vision, floaters, glare, and better vision in one eye. The most frequent symptoms for each group were light sensitivity (88%) in dry eye, better vision in one eye (62%) in glaucoma suspect and (94%) in glaucoma, and worsening vision (89%) in cataracts. Additionally, the symptoms that are more common in dry eye than any other group, and which occurred in more than half of dry eye patients, include glare (77%), floaters (73%), difficulty focusing (70%), vision that varies across the day (67%), halos (61%), and spots (56%). The seven symptoms that are more common in dry eye as compared to other groups is shown in Fig. 1, with significantly different distributions of frequency ratings (*p* < 0.05 for all) and severity ratings (*p* < 0.01) across disease groups.

### Univariate analysis of symptoms that distinguish dry eye from glaucoma suspects, glaucoma, and cataract

In univariate analysis, comparison of frequency and severity of symptom associations with dry eye versus another disease yielded similar significant associations across symptoms. Therefore, the primary analysis for multivariable analysis was frequency of symptoms, as this is inclusive of the presence of having symptoms of any severity (i.e. patients who report symptoms of any severity must also experience the symptom at least rarely).

### Symptoms distinguishing dry eye from glaucoma suspects

**Fig. 2:**
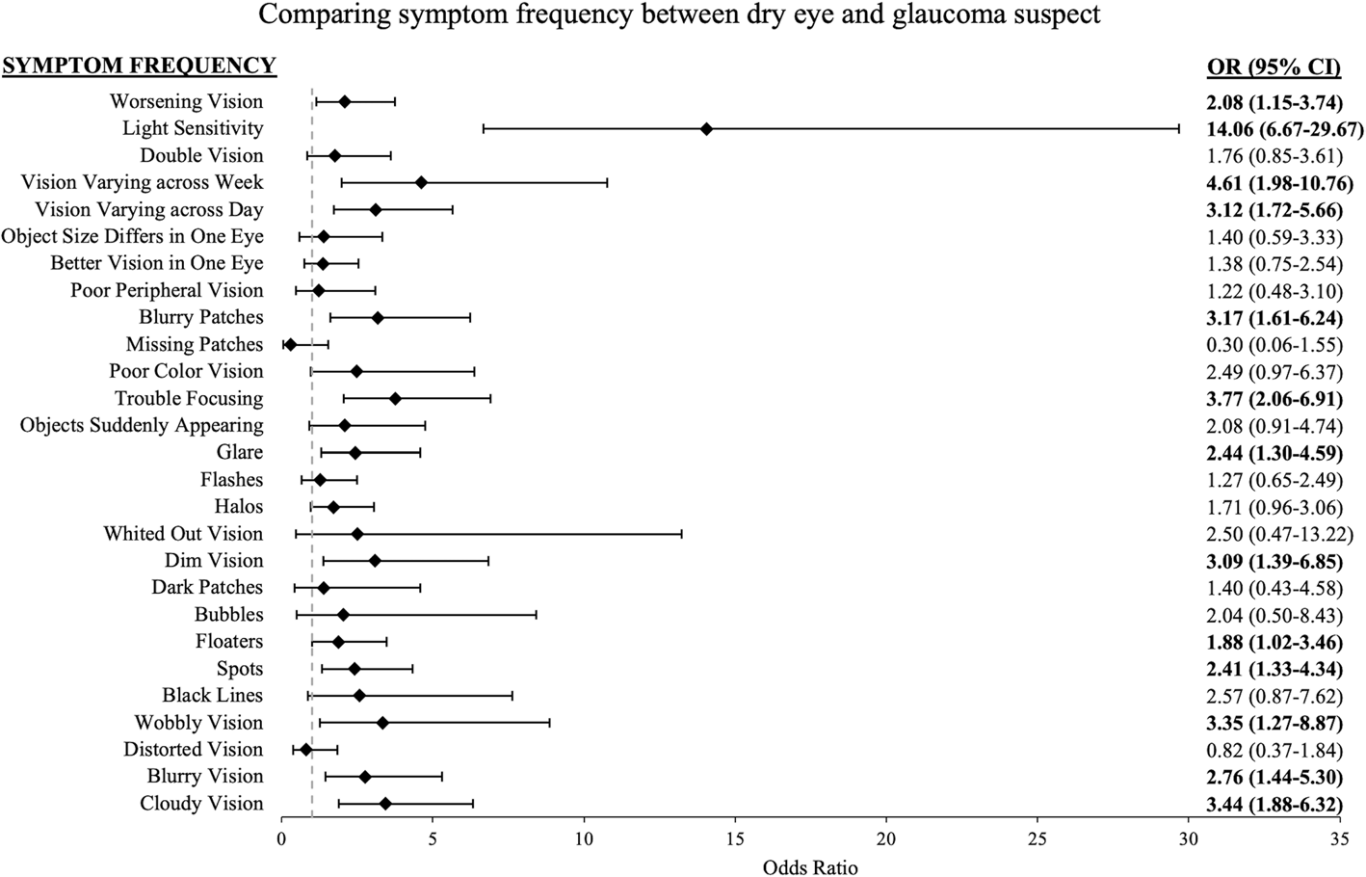
Univariate logistic regression models of dry eye versus glaucoma suspect symptom frequency. Odds ratio (OR) > 1 indicates symptom is more likely to occur in dry eye subjects compared to glaucoma suspect controls. *CI* = confidence interval

In univariate analysis, the odds of experiencing worsening vision, light sensitivity, vision that varies across the week, vision that varies across the day, blurry patches of vision, difficulty focusing, glare, dim vision, floaters, spots in vision, blurry vision, and cloudy vision were significantly higher in dry eye as compared to glaucoma suspects (Fig. 2). Based on the backwards selection, a multivariable model including frequency of light sensitivity and spots in vision, along with the demographic covariates (age, sex, race, education, and employment) best distinguished a diagnosis of dry eye from glaucoma suspect (Table 2). In these multivariable models, as compared to glaucoma suspect controls, dry eye patients had a significantly greater odds of reporting light sensitivity (OR = 14.95, 95% CI = 6.25–35.74) and spots in vision (OR = 2.75, 95% CI = 1.20–6.31). When this final multivariable model was compared to a nested model with only the demographic variables, the symptom of light sensitivity frequency contributed significantly to the model fit distinguishing the two diseases (*p* < 0.001, full model adjusted R^2^ = 0.32, nested model adjusted R^2^ = 0.13). An ROC curve constructed from the final model’s ability to predict a diagnosis of dry eye from glaucoma suspect had a cross-validated mean AUC of 0.84 (cross-validated SD = 0.067), with a sensitivity of 78% and specificity of 83%.

**Table 2:** Sets of visual symptoms best distinguishing dry eye from glaucoma suspect, glaucoma, and cataract

**Dry Eye vs. Glaucoma Suspect** ^a^Likelihood Ratio Test *p* < 0.001 ^b^Cross-Validated Mean AUC (SD) = 0.84 (0.067) ^c^Sensitivity = 78%; Specificity = 83%			
	*Odds Ratio*	*95% CI*	*P value*
Light Sensitivity	14.95	6.25–35.74	< 0.001
Spots in Vision	2.75	1.20–6.31	0.017
**Dry Eye vs. Glaucoma** Likelihood ratio Test *p* < 0.001 Cross-Validated Mean AUC (SD) = 0.93 (0.026) Sensitivity = 89%; Specificity = 87%			
	*Odds Ratio*	*95% CI*	*P value*
Light Sensitivity	9.19	2.03–41.68	0.004
Poor Peripheral Vision	0.21	0.059–0.72	0.013
Better Vision in One Eye	0.087	0.010–0.72	0.023
Patches of Vision Missing	0.055	0.009–0.33	0.001
**Dry Eye vs. Cataract** Likelihood Ratio Test *p* < 0.001 Cross-Validated Mean AUC (SD) = 0.93 (0.047) Sensitivity = 86%; Specificity = 86%			
	*Odds Ratio*	*95% CI*	*P value*
Spots in Visions	4.51	1.52–13.42	0.007
Vision that Varies Across the Week	4.67	1.23–17.72	0.024
Worsening Vision	0.096	0.025–0.37	0.001
Blindness	0.13	0.020–0.80	0.029

*AUC* area under the receiver operator curve, *SD* cross-validated AUC standard deviation

^a^Likelihood ratio test comparing a model with all symptom frequency variables remaining after backward stepwise selection including all demographic variables, and the nested model with only demographic variables

^b^Cross-validated mean AUC computed with fivefold split of data in the model of significant frequency variables remaining after backward stepwise selection with all demographic covariates

^c^Sensitivity and specificity of multivariable model to predict dry eye diagnosis over another study disease with predicted probability set to 50%

### Symptoms distinguishing dry eye from glaucoma

**Fig. 3:**
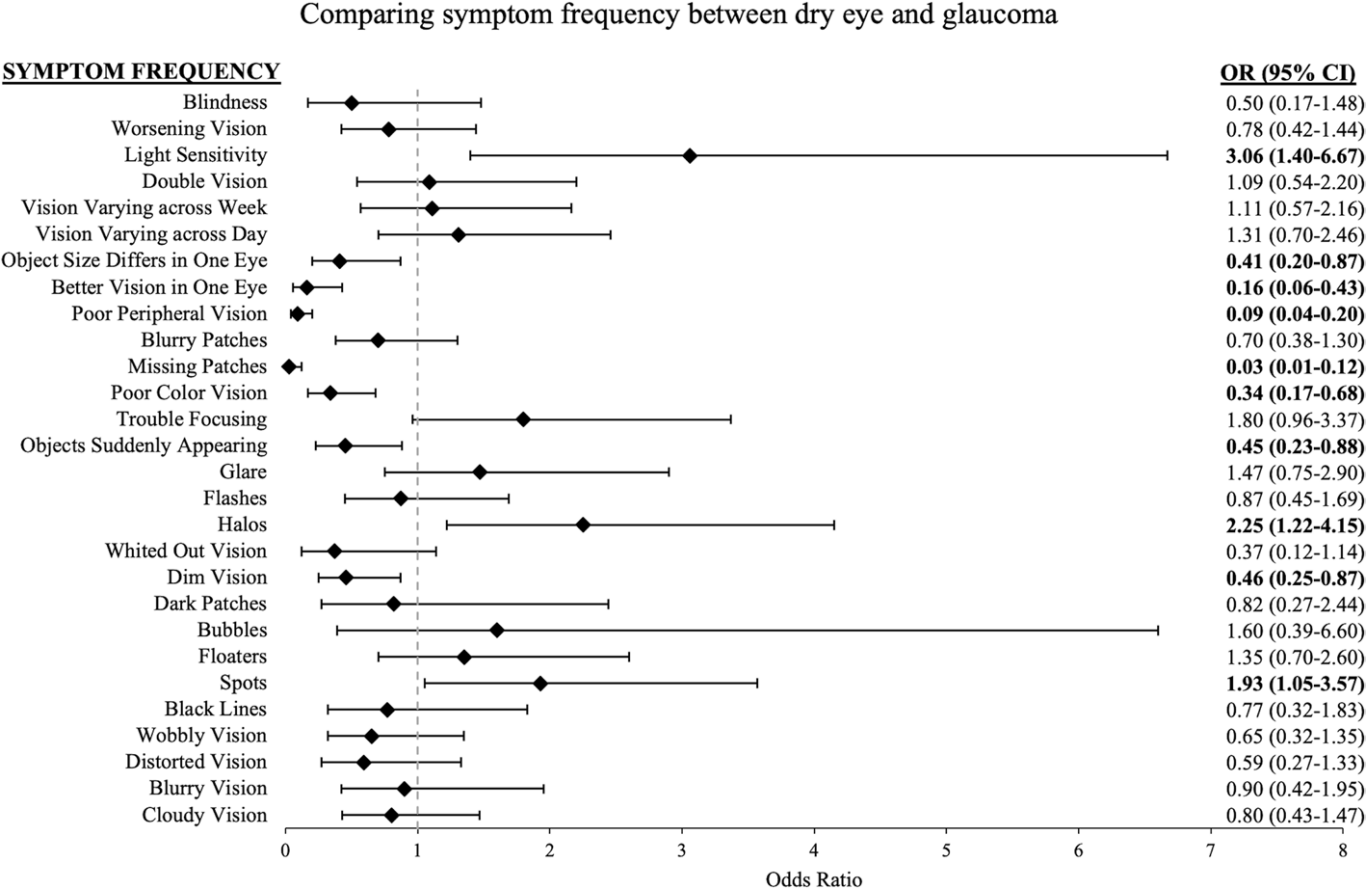
Univariate logistic regression models of dry eye versus glaucoma symptom frequency. Odds ratio (OR) > 1 indicates symptom is more likely to occur in dry eye diagnosis compared to glaucoma. *CI* = confidence interval

In univariate analysis, dry eye patients have significantly greater odds than those with glaucoma to experience light sensitivity, halos, and spots in vision, and have significantly lower odds to experience object size differing in one eye, better vision in one eye, poor peripheral vision, missing patches of vision, poor color vision, object suddenly appearing, and dim vision, (Fig. 3). Based on the backwards selection, a multivariable model of frequency of light sensitivity, worse peripheral vision, better vision in one eye, and patches of vision missing, along with demographic covariates age, sex, education, and employment, best distinguished dry eye from glaucoma (Table 2). In these multivariable models, compared to glaucoma patients, dry eye patients had significantly greater odds of experiencing light sensitivity (OR = 9.19, 95% CI = 2.03–41.68), but significantly lower odds of experiencing poor peripheral vision (OR = 0.21, 95% CI = 0.059–0.72), better vision in one eye (OR = 0.087, 95% CI = 0.010–0.72), and missing patches of vision (OR = 0.055, 95% CI = 0.009–0.33). When the final multivariable model was compared to a nested model with only the demographic variables, the symptom variables contributed significantly to the model’s ability to distinguish dry eye from glaucoma (*p* < 0.001, full model adjusted R^2^ = 0.53, nested model adjusted R^2^ = 0.37). The ROC curve constructed from the final model’s ability to predict a diagnosis of dry eye from glaucoma had a cross-validated mean AUC of 0.93 (cross-validated SD = 0.026), with a sensitivity of 89% and specificity of 87%.

### Symptoms distinguishing dry eye from cataract

**Fig. 4:**
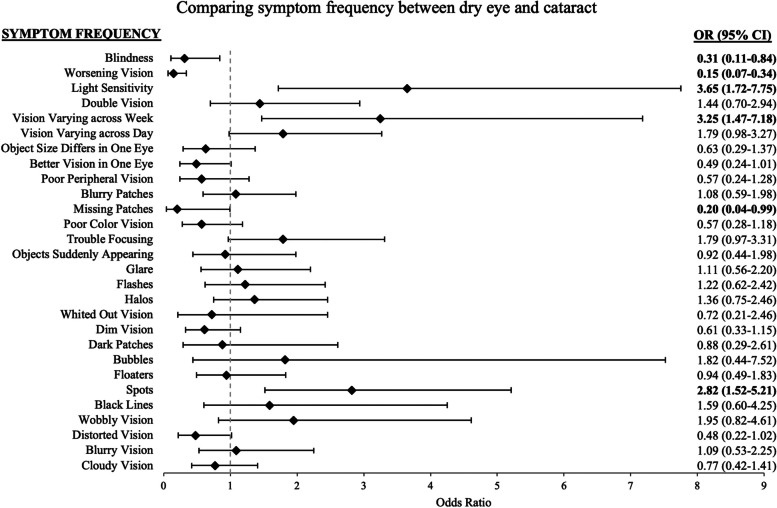
Univariate logistic regression models of dry eye versus cataract symptom frequency. Odds ratio (OR) > 1 indicates symptom is more likely to occur in dry eye diagnosis compared to cataract. *CI* = confidence interval

In univariate analysis, dry eye patients have significantly greater odds than cataract patients to experience light sensitivity, vision that varies across the week, and spots in vision, and have significantly lower odds to have blindness, worsening vision, and missing patches of vision (Fig. 4). Based on the backwards selection, a multivariable model of frequency of spots, vision that varies across the week, worsening vision, and blindness, best differentiated patients with dry eye vs. cataract (Table 2). In multivariable models, compared to cataract patients, dry eye patients had significantly greater odds of experiencing spots in vision (OR = 4.51, 95% CI = 1.52–13.42) and vision that varied across the week (OR = 4.67, 95% CI = 1.23–17.72), but significantly lower odds of describing worsening vision (OR = 0.096, 95% CI = 0.025–0.37) and or a feeling of blindness (OR = 0.13, 95% CI = 0.020–0.80). When this final multivariable model was compared to a nested model with only the demographic variables, the symptom variables contributed significantly to the model’s ability to distinguish dry eye from cataract (*p* < 0.001, full model adjusted R^2^ = 0.51, nested model adjusted R^2^ = 0.41). The ROC curve constructed from the final model’s ability to predict a diagnosis of dry eye from glaucoma had a cross-validated mean AUC of 0.93 (cross-validated SD = 0.047), with a sensitivity of 86% and specificity of 86%.

### Sensitivity analysis

A sensitivity analysis excluding all non-dry eye patients using a prescription dry eye treatment (three glaucoma suspect patients, two glaucoma patients, and four cataract patients) was conducted. In the multivariable logistic regression models, the same visual symptoms had significant associations in pariwise comparisons between dry eye and the other conditions as the multivariable logistic regression models with the full patient sets. Frequency of dim vision was an additional visual symptom that was significantly more likely to be reported in dry eye versus glaucoma suspect patients (OR 4.58, 95% CI 1.39–15.05).

## Discussion

In our study, patients with dry eye can be differentiated from those without significant eye disease (suspected glaucoma), glaucoma, and cataracts with reasonable sensitivity and specificity using sets of visual symptoms, though in the real world these symptoms would supplement careful clinical examination. The most common symptom was light sensitivity in dry eye patients, and the presence of light sensitivity helped differentiate dry eye from both glaucoma suspect controls and glaucoma patients, while other symptoms (i.e. spots in vision, varying vision), helped distinguish dry eye and cataract patients. Several nonspecific symptoms that did not help distinguish dry eye from other conditions were identified, including glare (which differs from light sensitivity by the absence of pain), floaters, difficulty focusing, vision varying across the day, and halos. Similar associations were observed for severity of symptoms in distinguishing dry eye from other conditions, which is also important from a patient perspective. These data can help guide the specific situations in which symptoms reported by patients can be used to help distinguishing dry eye from other ocular conditions.

Light sensitivity and spots in vision, both of which can be a result of tear film disturbance, best distinguished dry eye from relatively normally-sighted controls (glaucoma suspects). Visual disturbances like spots in vision may reflect the perception of an imperfect ocular surface leading to wavefront aberrations and light scattering [11, 32–35]. Superficial corneal lesions (measured by OSS) caused by dry eye can lead to light sensitivity through irritation of trigeminal afferents [12, 32]. In agreement with our findings, dry eye has been suggested to be the most common cause of light sensitivity [12].

Light sensitivity was also more common in dry eye patients as opposed to those with glaucoma, though additional symptoms more common in glaucoma could also differentiate the conditions. Partly consistent with prior work describing the symptoms most strongly associated with glaucoma severity in a glaucoma population, [20] we found that experiencing poor peripheral vision, better vision in one eye, and missing patches of vision differentiate glaucoma and dry eye patients. Indeed, these visual symptoms should only rarely occur in dry eye as visual problems should not vary across parts of the visual field. Notably, cloudy vision was non-specific and described at a similar frequency in both glaucoma and dry eye in our study, consistent with prior work showing cloudy vision symtpoms being more prevalent in dry eye related to contact lens wear [24, 33].

Light sensitivity could not distinguish dry eye from cataracts, as cataracts can also cause light sensitivity via light scattering, along with other common visual symptoms in cataracts including blurry vision, worsening vision, changes in color vision, halos, light sensitivity, and glare [34, 35]. We did identify other symptoms that could distinguish patients with cataract from dry eye, including worsening vision and feeling blind (more common in cataract patients), and spots in vision or vision that varies across the week are (more common in dry eye patients). With cataract, patients frequently complain of loss of visual acuity as a cataract progresses, [35] and with our study focusing on patients ready for cataract surgery, recent visual worsening would be common. Vision varying over the course of the week also differentiated dry eye from cataracts (but not other conditions), verifying the notion that cataract effects should be constant, while dry eye can affect vision differently on a short-term basis, [36] i.e. immediately after waking up or after prolonged screen time as a result of fluctuating frequency of blinking during computer-related work [36, 37].

This study provides data that can be used to incorporate patient-reported visual symptoms in the diagnosis of eye disease and patient communication. We highlight symptoms which can be relatively sensitive and specific in distinguishing dry eye from other common ocular conditions. Given the low and inconsistent associations between the symptoms and signs of dry eye disease, visual symptoms should be carefully considered even if objective measures do not strongly support dry eye [38]. For example, corneal neuropathy can occur in chronic dry eye and produce symptoms of dry eye such as light sensitivity even after the ocular surface findings of dry eye have mostly resolved [12]. Chronicity of dry eye or underlying disease may cause neurotrophic changes and these patients may not have typical dry eye symptoms such as burning, irritation, and pain but rather primarily light sensitivity and fluctuations in their vision [6]. However, using visual symptoms alone to diagnose ophthalmologic conditions is unlikely to be sufficient as visual symptoms common in dry eye are often non-specific, and should be interpreted in the context of physical exam tests when possible (i.e. to rule out other conditions) [39]. Notably, when physical exam data of the eye are not readily available (i.e. in non-ophthalmology offices, telehealth, low resource, or outreach settings), patient reported visual symptoms may be the primary information on which providers can make judgments on diagnosis and treatment. Additionally, symptoms can be iteratively assessed in follow up visits as new sets of symptoms may suggest the presence of advanced dry eye, cataract, or glaucoma, though not necessarily the progression of any study conditions. The data in our study is particularly useful in the context of distinguishing severe dry eye from *visually significant* glaucoma (judged by mean deviation cutoffs) or cataract (judged by the decision for surgery), reflecting the patients included in the study. Thus, our findings may not be useful in detecting early stages of disease as part of telemedicine visits or other similar settings. Symptoms still serve a purpose in ophthalmology clinics when an eye exam is possible; when a patient feels listened to, outcomes improve both in the form of accurate diagnosis and increased treatment adherence [40, 41].

This study has several limitations. The possibility of dry eye co-occurring in patients with the other conditions cannot be ruled out since the non-dry eye groups were not formally evaluated with regards to their ocular surface features. Indeed, it is not uncommon for dry eye to co-occur with glaucoma and cataracts, though the contribution of dry eye visual symptoms in the non-dry eye groups would have biased our findings towards the null. A sensitivity analysis excluding the non-dry eye patients taking medications that treat dry eye, though without a chart diagnosis of dry eye, yielded largely similar results except for the addition of dim vision being more likely reported in dry eye patients compared to glaucoma suspect patients. It is likely there was only subclinical dry eye remaining after the sensitivity analysis. There were patients within each non-dry eye group taking a prescription eye drop indicated for management of dry eye, though these only represented 3.5% of non-dry eye participants. Another limitation is the lack of granularity in our study with regards to severity/location of glaucoma damage, severity/type of cataract, and categorization of dry eye by objective measures other than OSS. As such, the set of symptoms with significant differences between dry eye and other conditions we found in our study may not help differentiate mild dry eye disease, early glaucoma, and non-visually significant cataract. Additionally, due to the COVID-19 pandemic, survey administration varied between groups as the survey was administered sequentially starting with glaucoma suspects and glaucoma patients in person before the pandemic, then cataract and dry eye patients by phone after the COVID-19 outbreak. The mode of administration may influence how patients answer the questions on visual symptoms. Also, symptom frequency was used in primary models for straightforward binarization of the data and because they have similar associations with the study condition comparisons as when symptom severity is used, though in clinical practice patients may be concerned about symptom severity. It is a limitation that we included dry eye patients under treatment to alleviate symptoms—though the fact that they required continued care in a dry eye clinic indicated they had chronic symptoms not fully relieved by therapy. Finally, the words people use to describe symptoms may vary across language, cultures, and country, limiting the generalizability of this work. Other demographic characteristics of this study population may also limit generalizability, such as the highly educated group of participants in this study across all disease groups that may not be representative of the education level of patients with these conditions in the general population.

## Conclusions

In conclusion, paying attention to the visual symptoms a patient experiences can help aid in the diagnosis of dry eye, glaucoma, or cataract in a variety of clinical settings, and also help make treatment decisions by understanding the most likely etiology of prevalent symptoms. Our study demonstrates that specific sets of symptoms may help differentiate significant dry eye from other conditions.

### Supplementary Information

**Additional file 1. **Visual symptoms questionnaire administered to study participants. The content of the questionnaire was derived from the Glaucoma Symptom Scale, Glaucoma Quality of Life (QoL) Bank, Eyetem Bank (QoL item for all eye diseases), and Cataract Symptom Score and administered by a trained researcher. 

## Data Availability

The datasets referenced in this study are not publicly available in order to best protect patient privacy but can be made available from the corresponding author upon reasonable request.

## Abbreviations

OSS: Ocular surface staining
TBUT: Tear break-up time
VA: Visual acuity
VF MD: Visual field mean deviation
SICCA: Sjögren’s International Collaborative Clinical Alliance
SITA: Swedish Interactive Testing Algorithm
logMAR: Logarithm of the minimum angle of resolution
AUC: Area under the curve
ROC: Receiver operator curve
SD: Standard deviation
OR: Odds ratio
CI: Confidence interval
