# Supplementary material for: Utilizing visual symptoms to distinguish dry eye from glaucoma, cataract, and suspect glaucoma patients: a cross-sectional study
Source: BMC Ophthalmol. 2024 Jan 9;24:16. doi: 10.1186/s12886-023-03219-2 (PMC10775660; doi:10.1186/s12886-023-03219-2)
Supplement: Supplementary file 1 — Additional file 1. Visual symptoms questionnaire administered to study participants. The content of the questionnaire was derived from the Glaucoma Symptom Scale, Glaucoma Quality of Life (QoL) Bank, Eyetem Bank (QoL item for all eye diseases), and Cataract Symptom Score and administered by a trained researcher. [file 12886_2023_3219_MOESM1_ESM.pdf]

## Demographic Questionnaire:

We would like to ask you some basic questions about yourself.

**DEM1. What is your date of birth?**

__ __ / __ __ / __ __ __ __
Month Day Year

**DEM2. Sex**

What is your sex? M F

**DEM3. Which of these groups best describes you?**

White …………………………………  1
African American ……………………  2
American Indian or Alaskan Native …  3
Asian or Pacific Islander ……………  4
Other ……………..…………………..  5

**DEM4. Is your main national ancestry Hispanic?**

(1) Yes (0) No

**DEM5. What was the highest grade in school that you completed?**

ELEMENTARY

No formal schooling ………………  0
1st Grade ………… ………………  1
2nd Grade ………… ………………  2
3rd Grade ………… ………………  3
4th Grade ………… ………………  4
5th Grade ………… ………………  5
6th Grade ………… ………………  6
7th Grade ………… ………………  7
8th Grade ………… ………………  8

HIGH SCHOOL

9th Grade ………… ………………...  9
10th Grade ………… ………………  10
11th Grade ………… ………………  11
12th Grade ………… ………………  12

COLLEGE AND GRADUATE SCHOOL

1 Year ………………………………  13

2 Years ……………………………… ☐ 14
3 Years ……………………………… ☐ 15
4 Years ……………………………… ☐ 16
Graduate or Professional School……. ☐ 17
Don't Know ……………………….. ☐ 99

**DEM6. Are you currently employed?**
(1) Yes (0) No

**DEM6a. If you answered Yes to the above question, which of the following best describes your employment?**
a. Full time …………………… ☐ (2)
b. Part time …………………… ☐ (1)
c. Occasional work / consultant ☐ (0)

**Medication List:**

We will now ask you about your use of prescription or over the counter eye drops. It will be helpful if you could show me all of the eye drops, prescription and nonprescription, that you are currently taking.

**Record the amount of medication subject is taking if different from the container.**
**If the subject is taking no medications, enter "None" as the first medication name.**

| A. What are the names of the medications? | B. How often are you supposed to take the medication? | | | C. Container seen? **(Observe)** | |
|---|---|---|---|---|---|
| Medication Name: | Every Day | As needed or less than once a day | Don't know | Yes | No |
| MEDN 1. | 1 | 2 | 99 | 1 | 0 |
| MEDN 2. | 1 | 2 | 99 | 1 | 0 |
| MEDN 3. | 1 | 2 | 99 | 1 | 0 |
| MEDN 4. | 1 | 2 | 99 | 1 | 0 |
| MEDN 5. | 1 | 2 | 99 | 1 | 0 |
| MEDN 6. | 1 | 2 | 99 | 1 | 0 |
| MEDN 7. | 1 | 2 | 99 | 1 | 0 |

| | | | | | |
|---|---|---|---|---|---|
| MEDN 8. | 1 | 2 | 99 | 1 | 0 |
| MEDN 9. | 1 | 2 | 99 | 1 | 0 |
| MEDN 10. | 1 | 2 | 99 | 1 | 0 |
| MEDN 11. | 1 | 2 | 99 | 1 | 0 |

**Vision Questionnaire:**

We will now transition to the visual symptom questionnaire. We are trying to determine what words patients used to describe their symptoms.

**VQ1. How long have you known about your ___________ (current eye condition)?**

**VQ2. On a normal day, describe how you see the world around you.**

**VQ3. How often do you feel the following symptoms? Please answer to what extent each of the following is an accurate representation of what you see.**

| | Frequency | | | | Severity | | | |
|---|---|---|---|---|---|---|---|---|
| | Never | Rarely | Sometimes | Very Often | Not At All | Mild | Moderate | Severe |
| | | | | | | | | |
| I feel like I'm looking through a cloud or fog. | 1 | 2 | 3 | 4 | 1 | 2 | 3 | 4 |
| My vision is blurry. | 1 | 2 | 3 | 4 | 1 | 2 | 3 | 4 |
| My vision is distorted. For example, lines that should be straight are curved or misshapen. | 1 | 2 | 3 | 4 | 1 | 2 | 3 | 4 |
| My vision is wobbly. | 1 | 2 | 3 | 4 | 1 | 2 | 3 | 4 |
| I see black lines in my vision. | 1 | 2 | 3 | 4 | 1 | 2 | 3 | 4 |
| I see spots in my vision. | 1 | 2 | 3 | 4 | 1 | 2 | 3 | 4 |
| I see floating objects in my vision. For example, things that look like specks, strings, cobwebs drift across my vision. | 1 | 2 | 3 | 4 | 1 | 2 | 3 | 4 |
| I see bubbles in my vision. | 1 | 2 | 3 | 4 | 1 | 2 | 3 | 4 |
| I see a gray or dark patch in the center of my vision. | 1 | 2 | 3 | 4 | 1 | 2 | 3 | 4 |
| I feel like my vision is dim/dark. | 1 | 2 | 3 | 4 | 1 | 2 | 3 | 4 |
| I feel like my vision is whited out. | 1 | 2 | 3 | 4 | 1 | 2 | 3 | 4 |

| | | | | | | | | |
|---|---|---|---|---|---|---|---|---|
| When I look at lights, I see halos around them. | 1 | 2 | 3 | 4 | 1 | 2 | 3 | 4 |
| I see flashes of light when I am not supposed to. | 1 | 2 | 3 | 4 | 1 | 2 | 3 | 4 |
| When I look at lights, there is a glare. | 1 | 2 | 3 | 4 | 1 | 2 | 3 | 4 |
| When I look out, objects will suddenly appear when I did not notice them before. | 1 | 2 | 3 | 4 | 1 | 2 | 3 | 4 |
| When I look at an object, it takes time for my eyes to focus. | 1 | 2 | 3 | 4 | 1 | 2 | 3 | 4 |
| When I look at certain colors, I cannot tell them apart. | 1 | 2 | 3 | 4 | 1 | 2 | 3 | 4 |
| Patches of my vision are missing. | 1 | 2 | 3 | 4 | 1 | 2 | 3 | 4 |
| Patches of my vision are blurred. | 1 | 2 | 3 | 4 | 1 | 2 | 3 | 4 |
| My vision is best at the middle of my eye with little vision at the periphery. | 1 | 2 | 3 | 4 | 1 | 2 | 3 | 4 |
| One of my eyes has better vision than the other. | 1 | 2 | 3 | 4 | 1 | 2 | 3 | 4 |
| When I look at an object with one eye, it looks bigger or smaller compared with the other eye. | 1 | 2 | 3 | 4 | 1 | 2 | 3 | 4 |
| My vision varies across times of the day. | 1 | 2 | 3 | 4 | 1 | 2 | 3 | 4 |
| My vision varies across times of the week. | 1 | 2 | 3 | 4 | 1 | 2 | 3 | 4 |
| I see double. | 1 | 2 | 3 | 4 | 1 | 2 | 3 | 4 |
| I am sensitive to light. | 1 | 2 | 3 | 4 | 1 | 2 | 3 | 4 |
| My vision is getting worse. | 1 | 2 | 3 | 4 | 1 | 2 | 3 | 4 |
| At certain times, I feel like I am blind. | 1 | 2 | 3 | 4 | 1 | 2 | 3 | 4 |
